# Modeling Algal Toxin Dynamics and Integrated Web Framework for Lakes

**DOI:** 10.3390/toxins17070338

**Published:** 2025-07-03

**Authors:** Özlem Baydaroğlu, Serhan Yeşilköy, Anchit Dave, Marc Linderman, Ibrahim Demir

**Affiliations:** 1 NOAA, Global Systems Laboratory, Boulder, CO 80305, USA; 2 National Academies of Sciences, Engineering, and Medicine, Washington, DC 20001, USA; 3 İstanbul Provincial Directorate of Agriculture and Forestry, Ministry of Agriculture and Forestry, İstanbul 34724, Türkiye; serhan.yesilkoy@usda.gov; 4 Oak Ridge Institute for Science and Education, Oak Ridge, TN 37830, USA; 5 USDA-ARS, Adaptive Cropping Systems Laboratory, Beltsville, MD 20705, USA; 6 IIHR–Hydroscience & Engineering, University of Iowa, Iowa City, IA 52242, USA; anchit-dave@uiowa.edu; 7 Department of Geographical and Sustainability Sciences, University of Iowa, Iowa City, IA 52245, USA; marc-linderman@uiowa.edu; 8 River-Coastal Science and Engineering, Tulane University, New Orleans, LA 70118, USA; idemir@tulane.edu; 9 ByWater Institute, Tulane University, New Orleans, LA 70118, USA

**Keywords:** harmful algae, microcystin, sparse identification of nonlinear dynamics (SINDy), environmental health, public health, web framework

## Abstract

Harmful algal blooms (HABs) are one of the major environmental concerns, as they have various negative effects on public and environmental health, recreational services, and economics. HAB modeling is challenging due to inconsistent and insufficient data, as well as the nonlinear nature of algae formation data. However, it is crucial for attaining sustainable development goals related to clean water and sanitation. From this point of view, we employed the sparse identification nonlinear dynamics (SINDy) technique to model microcystin, an algal toxin, utilizing dissolved oxygen as a water quality metric and evaporation as a meteorological parameter. SINDy is a novel approach that combines a sparse regression and machine learning method to reconstruct the analytical representation of a dynamical system. The model results indicate that MAPE values of approximately 2% were achieved in three out of four lakes, while the MAPE value of the remaining lake is 11%. Moreover, a model-driven and web-based interactive tool was created to develop environmental education, raise public awareness on HAB events, and produce more effective solutions to HAB problems through what-if scenarios. This interactive and user-friendly web platform allows tracking the status of HABs in lakes and observing the impact of specific parameters on harmful algae formation.

## 1. Introduction

Reduced water clarity, unpleasant odors and tastes, the proliferation of harmful algal blooms (HABs), the loss of aquatic animal populations, increased nutrient concentrations in primary producers, acidification, deoxygenation and shifts in the aquatic food web are all results of eutrophication, which is caused by an influx of nutrients like fertilizers or pollutants [1,2,3]. HABs are caused by many sources, such as water pollution based on agricultural activities, wastewater treatment plant discharges, leakages from sewer systems, natural factors like pH and light levels, and climate change impacts. In recent decades, HABs have been seen as a serious hazard to the environment, according to the consensus of the scientific community [2,3,4]. They have several detrimental effects on the environment [5,6] such as toxin accumulation in reservoirs or water bodies [7], the public, and economics [4,8].

The impact of climate change on HABs is anticipated to manifest in alterations to their frequency, magnitude, biogeographical distribution, phenology [9,10], and toxicity [11]. Generally, nutrient pollution from agriculture and industry, water temperature, and water quality parameters are the main drivers of HABs occurrence [5,12,13]. The Intergovernmental Panel on Climate Change (IPCC) Special Report on the Ocean and Cryosphere in a Changing Climate (SROCC), which was approved in September 2019, was the first time the connection between HABs and climate change was stated in a formal way.

HABs primarily consist of one or more species of cyanobacteria, commonly referred to as blue-green algae, including Microcystis and Anabaena [14]. Microcystins, predominantly generated by *Microcystis* spp., are the most widespread cyanobacterial toxins in global freshwater systems [15].

HAB researchers have endeavored to predict HAB indicators through statistical, process-based, and hybrid models [11,16,17]. [18] employed a generalized additive model that utilized an identity-link function for Gaussian distribution. The model incorporated diverse environmental variables such as sunspot numbers, winter North Atlantic Oscillation (NAO) indices, monthly mean rainfall, air and sea water temperature, salinity, winds and Ekman transport, and phytoplankton data. Using cyanobacteria biomass as an indicator, [19] used Bayesian network (BN) [20] to relate future climate change and land-use management scenarios to ecological state. [21] utilized empirical dynamic modeling (EDM) [22] to predict chlorophyll-a, demonstrating the efficacy of dynamic models in forecasting ecological parameters.

Some studies reveal that the frequency, volume, biogeography, phenology, and toxicity of HABs are likely to vary as a result of climate change [11,23,24,25,26,27]. Increased ocean stratification [28,29] brought on by greater glacier melting, higher air temperatures [30,31], changing precipitation [32,33] and wind patterns [34,35,36], changed nutrient availability and composition [37], light intensity [33], and ocean acidity all have an impact on HABs [11]. Furthermore, the dispersion of HABs can be influenced by wind, air and lake temperature [38], while precipitation can facilitate the introduction of nutrients into aquatic environments, thereby promoting the development of HABs [6].

There were various efforts to study HAB events, observed in the state of Iowa, which heavily relies on agriculture as its primary economic sector [39,40]. In their study, ref. [41] developed a novel metric for normalizing microcystin congeners, enabling a comparative analysis of water bodies impacted by cyanobacterial harmful algal blooms (CyanoHAB). Additionally, they introduced a geometry-based image processing technique that facilitated the integration of aerial images captured by a drone, taken from above the water surface. A significant linear relationship was observed between the concentrations of chlorophyll-a and microcystin in lakes located in Iowa, as evidenced by a correlation coefficient of 62%. The researchers also observed that the feasibility of multispectral imaging for estimating microcystin concentrations may be limited at present, primarily due to the spectral constraints of the multispectral camera. Ref. [42] conducted a collection of 65 water samples from various lake beaches in Iowa to examine the potential relationship between the concentrations of microcystin and the abundances of genes responsible for toxin production. Strong correlations were observed between the abundance estimations of mcyA genes and microcystin concentrations in lake water samples. In a study conducted by [41], it was discovered that microcystins were present in all 10 lakes that were sampled in Iowa. Furthermore, microcystin was identified as the predominant toxin in 90% of these samples. Ref. [43] carried out a study where they collected water samples from 38 lakes in Iowa from 2018 to 2021. They developed three models using nine variables, which included chemical, biological, climatic, and land-use factors, to predict cyanobacterial HABs for a one-week period.

HABs modeling is a challenging task due to following reasons: (1) it is affected by various and multidimensional factors [44,45]; (2) HABs show complex nonlinear behavior [21,36,46]; (3) they are not uniform both in time and space [47,48]; and (4) there is not sufficient and continuous data [41,49,50]. Therefore, existing physical models have some difficulties [51] to find relationships between each factor affecting HABs prediction and many variable parameters should be required. It is costly and time-consuming to get around these restrictions.

Sparse Identification of Nonlinear Dynamics (SINDy) [52] employs sparsity methodologies and machine learning algorithms to reveal the differential equations that govern a dynamical system. It exploits the observation that the majority of dynamical systems exhibit a limited number of significant terms. This method utilized in various applications such as simulating and optimizing microalgal and cyanobacterial photo-production processes [53], physics-informed learning [54], predicting blood glucose levels [55], modeling air pollutants [56].

To overcome HABs’ modeling challenge, SINDy was used to model microcystin, which is one of the main indicators of HABs, using dissolved oxygen and daily total evaporation. We selected dissolved oxygen as the water quality criterion. Furthermore, it is the water quality parameter that has the highest amount of accessible data. Another factor included in the study is evaporation, which is a combination of a set of atmospheric variables. This analysis also incorporated other meteorological characteristics, including wind speed, maximum air temperature, lake water mixed layer temperature, and precipitation. Since there is a correlation between all the above meteorological characteristics, it is crucial to incorporate one of these elements into the modeling process to ensure precise modeling. The SINDy allows us to model HAB formation with discrete input dataset [57] and identify the governing equations that underlie nonlinear natural phenomena [52].

In order to effectively communicate the drivers and impacts of the HAB model, however, it is necessary to integrate such predictive models with web-based technologies. Widespread use of these web-based tools for data sharing, scientific visualization, data analytics, monitoring critical parameters, dissemination of necessary warnings, and decision support. The development of these information systems [58] is extremely beneficial for enhancing social awareness [59] in terms of scientific communication [60]. As previously mentioned, it has been determined that the state of Iowa has had a severe HAB problem in recent years, and the public’s awareness of this significant environmental issue falls short of expectations [61]. Therefore, it is imperative to disseminate information and enhance public awareness on the issue of HABs in lakes across Iowa.

To address this requirement, we created a web-based interactive communication tool, which includes the algal toxin, microcystin, model based on SINDy for selected lakes in Iowa. This tool has been developed to share the results, estimate the condition of the lakes according to what-if scenarios, increase awareness about HABs, and help decision-making mechanisms. In addition, it provides an easily accessible mapping environment (e.g., Google Maps API) on the web. This web platform may be used not only by water professionals but also by teachers, students and the public. When users change any variable, they will be able to see for themselves the change in harmful algae formation in the lake and determine whether the harmful algae value in the lake remains within the safe range for swimming, fishing, etc.

This paper is structured as follows: Section 2 explains the study area, data, SINDy method, and educational framework of HABs in some Iowa lakes. The results of the HAB modeling and its integration into the web-based information system can be found in Section 3. Some suggestions and evaluations were given in Section 4.

## 2. Results and Discussions

### 2.1. SINDy Model

**Figure 1:**
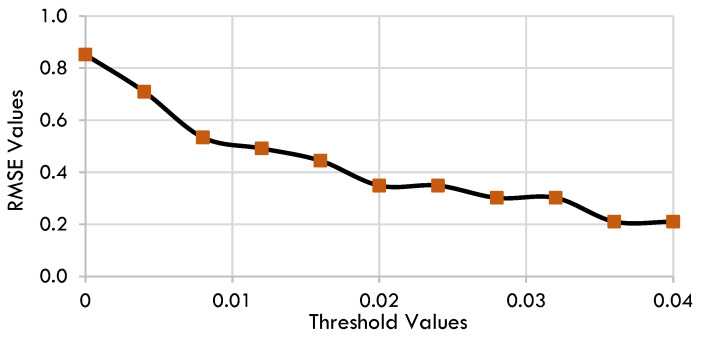
RMSE values vs. threshold values for the model construction for pre-processed data.

The analyzed data is partitioned into training data, which accounts for 75% of the total, and test data, which accounts for the remaining 25%. Gaussian noise with a standard deviation of 10% of the root mean square error (RMSE) was added to the training data, ensuring that only the most significant terms were retained in the model. The subset of candidate terms in the system was determined using sequential thresholded least squares (STLSQ) as an optimizer since the SINDy algorithm, in its standard form, utilizes the STLSQ method. The algorithm is specifically designed for the least squares formulation and performs effectively, although it lacks the ability to easily incorporate modifications such as extra constraints, resilient formulations, or nonlinear parameter estimates [62]. The model was fitted to the noisy data, and the coefficients were stored in an array. The performance of the model was assessed using test data. It is crucial to note that as the threshold increases, the model includes fewer terms, making it sparser and reducing the risk of overfitting to noise. Nevertheless, setting the threshold too high can potentially remove crucial dynamics. Hence, the optimal threshold value is being sought for promoting sparsity. Figure 1 illustrates the relationship between RMSE in the test data and threshold values on the testing trajectory of dM/dt where M indicates microcystin. The optimal threshold value is the value that minimizes the RMSE while preserving significant terms. Put simply, the optimal threshold value is the one that effectively captures important dynamics and does not overfit with noise.

**Figure 2:**
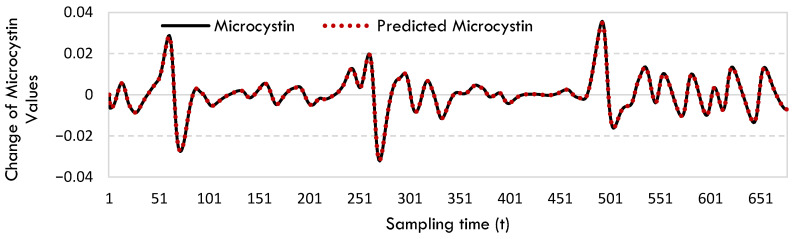
The rate of change in microcystin and predicted microcystin values for West Okoboji Lake.

**Figure 3:**
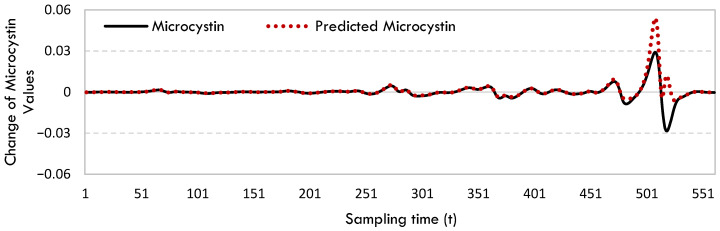
The rate of change in microcystin and predicted microcystin values for McIntosh Woods Lake.

**Figure 4:**
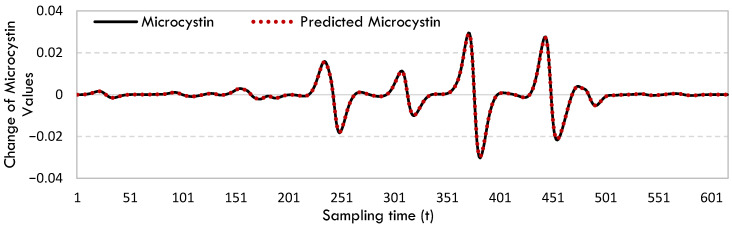
The rate of change in microcystin and predicted microcystin values for Blackhawk Lake.

**Figure 5:**
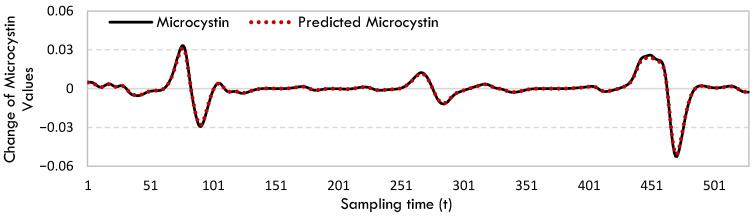
The rate of change in microcystin and predicted microcystin values for Geode Lake.

West Okoboji, McIntosh Woods, Black Hawk, and Geode Lake were chosen for modeling microcystin using dissolved oxygen and evaporation factors with SINDy. Details of threshold selection and equations for microcystin, dissolved oxygen, and evaporation are provided for the West Okoboji Lake. Only the final microcystin equations are given for the other lakes. The data presented in Figure 2, Figure 3, Figure 4 and Figure 5, which display the microcystin and predicted microcystin graphs for each lake, were not retrieved prior to pre-processing as they illustrate the rates of change in the microcystin levels. It is evident that performing such a procedure will elevate the error rates.

*West Okoboji Lake*

The optimal threshold value was determined to be 0.038 for West Okoboji datasets. Figure 1 displays RMSE values plotted against the threshold values for constructing the model using these datasets.

The equation system for microcystin (M), dissolved oxygen (D), and evaporation (E), determined using the optimal threshold value, can be represented as follows (Equations (1)–(3)):

(1) $$\frac{dM}{dt}=0.984M-2.352MD-0.464ME$$

(2) $$\frac{dD}{dt}=0.121ME$$

(3) $$\frac{dE}{dt}=0.114M-0.054D-0.281MD+0.099D^{2}+0.069DE-0.058E^{2}$$

The rate of change in microcystin data was calculated by integrating these equations. Figure 2 displays the rate of change in microcystin and the projected microcystin values for West Okoboji Lake.

Figure 2 demonstrates that the SINDy model accurately predicts this change with exceptional accuracy, especially when the microcystin change is very sharp.

*McIntosh Woods Lake*

SINDy gives rise to the model presented in Equations (4)–(6) for McIntosh Woods Lake.

(4) $$\frac{dM}{dt}=3ME$$

(5) $$\frac{dD}{dt}=-0.065+0.539M+0.337E-3.105M^{2}-0.356MD-0.775ME+0.145D^{2}-0.241DE-0.328E^{2}$$

(6) $$\frac{dE}{dt}=0.33+12.97M-2.32D+0.5E-90.64M^{2}-0.7MD-24.82ME+2.74D^{2}+1.23DE-0.5E^{2}$$

The model developed by SINDy identified a significant number of terms, potentially indicating that the approach referenced produces a model of the current system that lacks generalizability. Figure 3 displays the rate of change in microcystin and the projected microcystin values for McIntosh Woods Lake.

The microcystin change rate in McIntosh Woods Lake has remained constant over an extended period of time. It was observed that this value increased rapidly towards the end of the time period. Although the forecast model accurately predicted this sudden rise, it appears to have overestimated it.

*Blackhawk Lake*

The model for McIntosh Woods Lake is derived from SINDy and is represented by Equations (7)–(9).

(7) $$\frac{dM}{dt}=0.9828M-1.08MD-1.5732ME$$

(8) $$\frac{dD}{dt}=-0.133M^{2}$$

(9) $$\frac{dE}{dt}=-0.097M^{2}-0.124ME$$

Figure 4 depicts the rate of changes in microcystin and the predicted microcystin values for Blackhawk Lake.

While accurately predicting variations in change is challenging, the SINDy model effectively captures fluctuations in the rate of change.

*Geode Lake*

The equation system (Equations (10)–(12)) for Geode Lake is as follows:

(10) $$\frac{dM}{dt}=0.952M-0.356MD-1.688ME$$

(11) $$\frac{dD}{dt}=0.072D+0.029E+0.301M^{2}-0.257DE-0.022E^{2}$$

(12) $$\frac{dE}{dt}=0.188M-0.372ME$$

Figure 5 displays the rate of change in microcystin and the projected microcystin values for Geode Lake.

The estimations for Geode Lake are comparable to those conducted for other lakes. It is seen that the model accurately predicts times of rapid increase or decrease in rate of change values. The prediction outcomes for lakes have demonstrated that the forecasts generated by SINDy are highly effective in predicting the time periods during which harmful algae experience rapid growth or decline. Table 1 shows the prediction model performance results for every lake. Correlation coefficient (r), root mean square error (RMSE) and mean absolute percentage error (MAPE) are used as performance indicators.

The correlation coefficients between model findings and observations in lakes other than McIntosh Woods are highly proximate to 1. The reason for this is that SINDy perfectly captured the observed values for all lakes but McIntosh Woods. The prediction findings for McIntosh Woods Lake are satisfactory, albeit its prediction accuracy is lower compared to other lakes. The MAPE results indicate that the SINDY model effectively forecasts the fluctuations in nonlinear microcystin data.

### 2.2. HALGIS Web Framework

**Figure 6:**
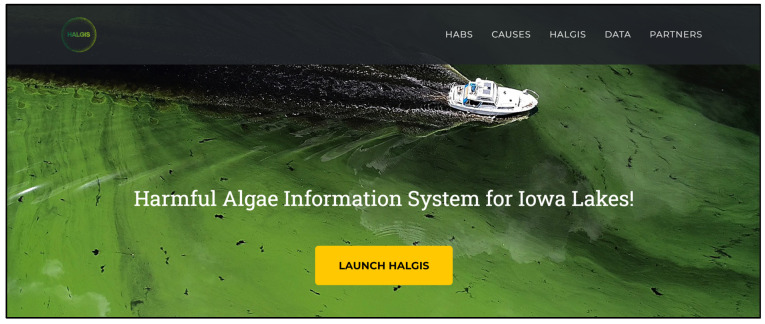
Harmful Algae Information System (HALGIS) landing page.

**Figure 7:**
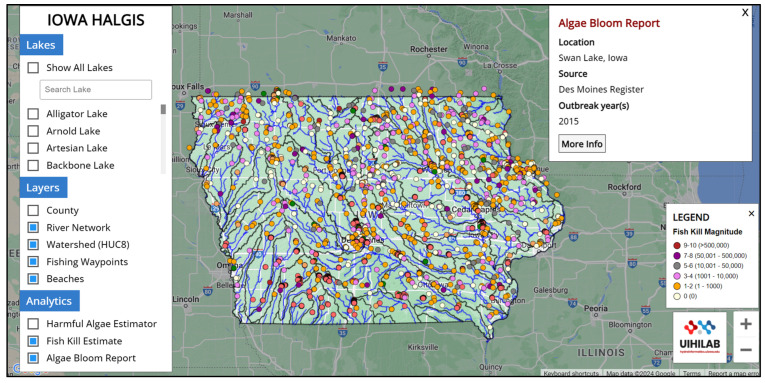
This web framework also allows their users to select different lakes and provides HAB-related information about beaches, fishing waypoints, fish kill estimates, and algae bloom reports, which were integrated from the Iowa Department of Natural Resources databases.

**Figure 8:**
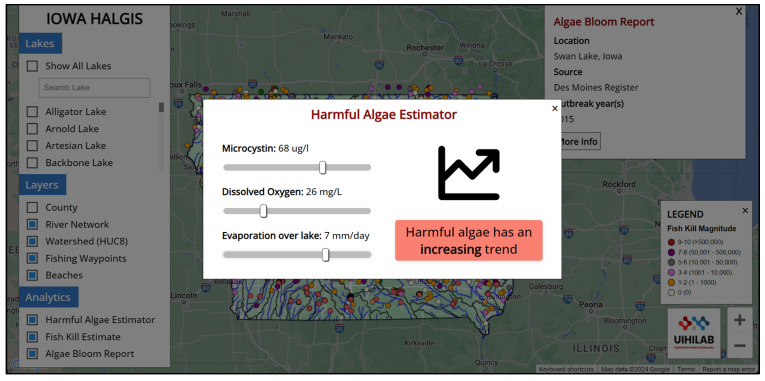
SINDy-based algae trend modeling can be calculated through sliders for the dissolved oxygen and evaporation rate over the lake. Sliders allow users to change the harmful algae-related factors and calculate the harmful algae trends over the lake.

HALGIS is a publicly available informational web platform (Figure 6) that can be accessed freely at https://hydroinformatics.uiowa.edu/lab/halgis (accessed on 2 July 2025). The landing page contains details on the datasets utilized and the analysis available in the system. These harmful algae ML-based prediction results based on SINDy and environmental factors were incorporated into the HALGIS. The data obtained from multiple sources will be temporarily saved in a local database. The web platform incorporates the Google Maps API to display GeoJSON files of the selected lakes in the study area (Figure 7). This allows users to see the size of the lake and which river network and watershed (HUC-8 level) it is connected to. Users are able to open the harmful algae estimator module and change the environmental variables (microcystin, dissolved oxygen, and evaporation) to see the harmful algae trend for the West Okoboji Lake (Figure 8).

HALGIS elevates the understanding of environmental sustainability among different user groups. The general public can utilize it as an informational guide to assess the quality of their nearby lakes, assisting in promoting local ecological awareness and engagement. For educators, it provides a dynamic, interactive tool that promotes in-depth exploration and understanding of aquatic ecosystems and the influence of environmental factors. Students, particularly those involved in environmental science programs, can use HALGIS as a substantial research tool, leveraging authentic data to practice and refine their research skills. The interactivity offered by the platform fosters proactive learning and encourages users to think critically about the interrelatedness of environmental factors and their effect on our water bodies. Thus, HALGIS proves to be a remarkable asset in fostering a more informed and environmentally conscious society.

The HAB estimator indicates a positive correlation between the rise in microcystin levels and the occurrence of HAB events in the lake. Furthermore, it is possible to analyze not only the presence of microcystin but also the comprehensive changes in dissolved oxygen and evaporation parameters, as well as the variations in HAB occurrences in the lake. Displaying the interactive HAB trend would enhance users’ knowledge of this environmental concern and improve the communication abilities of environmental science students as well as the educators.

## 3. Conclusions

Environmental contaminants and climate change can lead to the development of harmful algal blooms (HABs) in lakes, affecting ecological balance. These formations in lakes can grow to such an extent that they endanger the survival of other organisms in the environment and pose a risk to public health by contaminating drinking water sources. This study aimed to simulate HABs, a critical aspect for environmental health. For this objective, all water quality parameters linked to HABs, indicators of harmful algal presence in the lake, and pertinent meteorological factors were analyzed. Data availability is the main focus in these assessments. As is known, the primary issue in HAB investigations is the insufficient data availability. The second issue that needs to be addressed is synchronizing the data for these parameters. For instance, one water quality measurement could be recorded within an hour, whereas another one could be measured at a different day or time. After identifying various discrepancies, a comprehensive set of data combinations was established, and multi-dimensional time series were generated by aligning them with relevant meteorological data. These time series were used to model HABs with SINDy.

Multiple reasons influenced the selection of SINDy for HAB modeling. The SINDy approach is chosen for its exceptional modeling capability, which remains effective even with limited data. Additionally, it demonstrates robustness in handling data noise features and is well-suited for discrete data. These advantages have been highlighted in research conducted by [57,63,64,65,66]. As a result of modeling experiments, microcystin (a toxic substance produced by harmful algae), dissolved oxygen (a water pollution parameter), and evaporation (a meteorological variable containing temperature and precipitation information) were selected as the three variables that gave the sparsest equation to be used in the study.

The primary lake in the study is West Okoboji Lake which is used actively for various recreational activities such as boating, swimming, and water skiing. The graphs based on the microcystin equations’ results derived from SINDy (Figure 1, Figure 2, Figure 3 and Figure 4) reveal the following about the lake. The equations derived for all lakes did accurately represent the numerical change in microcystin; they precisely described the variations in microcystin values. The high correlation and quite low error values in Table 1 confirm this observation. The SINDy method accurately predicted the nonlinearly varying toxin microcystin, which is produced by cyanobacteria. All models created by SINDy for all lakes share the common characteristic of having a strong capacity to forecast extreme points, in contrast to conventional prediction models.

HALGIS web platform was developed as an information system with integrated data access, analysis, and visualization capabilities. HALGIS is a comprehensive online platform that provides access to harmful algae conditions, HABs-related data, information, and interactive visualizations. HALGIS offers information on monitoring harmful algal blooms and the real-time condition of lakes, while also serving as an educational tool on environmental pollution. Students can acquire insight into future HABs generation by adjusting parameter values and will have the ability to observe the climate change impact on environmental sustainability.

To address the data issue, crucial for future HAB studies, it is essential to standardize data collection by ensuring all measurements are taken simultaneously in a uniform format. Validation with ground-based data is essential for the wider utilization of satellite datasets, highlighting the important nature of the data collection step. Benchmark datasets following FAIR (findability, accessibility, interoperability, and reusability) data principles should be created and shared to tackle the significant threat to environmental health issues posed by HABs. Benchmark datasets may enhance estimation and prediction studies on harmful algae by granting access to the latest data. As research progresses, understanding of climate change and its effects on HABs increases, allowing for more precise planning of preventative and protective actions. Advancements in information systems for lake ecosystems and HABs will allow for real-time monitoring of lake pollutants and environmental health.

## 4. Materials and Methods

### 4.1. Study Area

**Figure 9:**
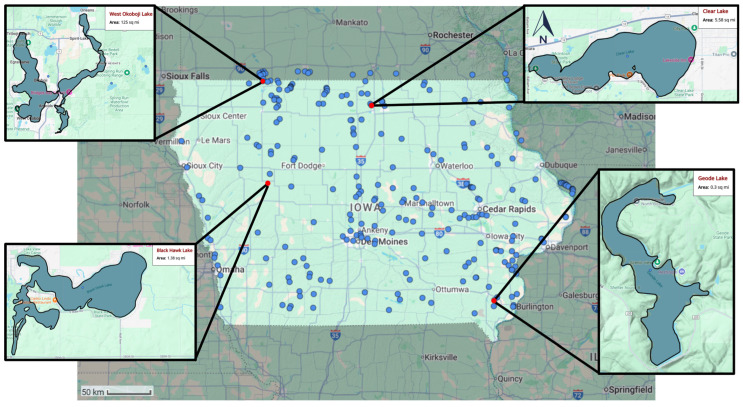
Study Area, which covers the most HAB-experienced lakes in the State of Iowa.

In recent decades, Iowa’s lakes have experienced the expansion of cyanoHABs distribution [41,61]. The existing monitoring of cyanoHABs in Iowa is insufficient, resulting in a paucity of data on specific microcystin congeners [41]. West Okoboji, McIntosh Woods (Clear Lake), Black Hawk, and Geode Lakes (see Figure 9) that had the most easily obtainable data were chosen as the pilot lakes for the study. These lakes are significant due to their comparatively larger surface area, proximity to rivers, and regular utilization by the public for sports and recreational pursuits, including fishing (with a habitat for over 25 fish species), swimming, camping, and boating. In Figure 9, blue lines, blue dots, and red dots denote rivers, lakes, and selected lakes, respectively.

### 4.2. Case Study

The study analyzed various water quality parameters, including dissolved oxygen, chlorophyll-a, total phosphorus, total nitrogen, microcystin, pH, and turbidity data of the lakes, to identify indicators of harmful algal blooms from the Iowa Department of Natural Resources AQuIA database. The study was unable to use every variable due to the unfixed sampling intervals (7 days, 8 days, 10 days or 14 days, etc.) and the very small and discontinuous number of data points for some parameters. After considering the availability and consistency of the data, it was determined that microcystin and dissolved oxygen data would be used.

**Figure 10:**
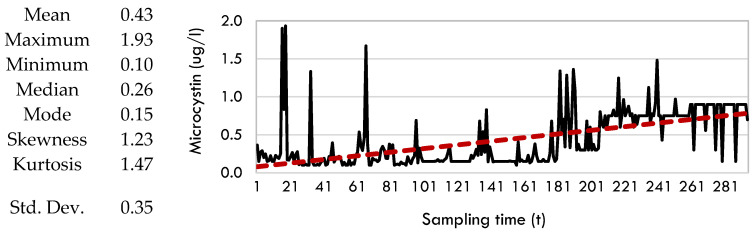
Raw microcystin data for Iowa and its summary statistics between the years 2006 and 2022 and the months May and September.

The time range of algal data is limited to the period from May to September due to certain meteorological and lake water conditions that promote algae development. The primary challenge encountered during the investigation was the acquisition of adequate data at consistent intervals. West Okoboji Lake was designated as the primary lake due to its ample size and form, which allow for data collection from multiple observation sites. The data for West Okoboji was collected from the stations listed in Table 2. Data for additional lakes were obtained at the specific sample site of each corresponding lake. Figure 10 presents the statistical information and graphical representations of the microcystin data. The trend line (red dashed line) in Figure 10 clearly illustrates the rise in microcystin values.

In addition, ECMWF Reanalysis hourly ERA5-land data, which are the latest global reanalysis data from 1950 to present with 0.1° spatial horizontal resolution, were used as meteorological data. The meteorological data used in the study were hourly wind speed at 2 m, air temperature, evaporation, lake mixed layer temperature, and precipitation data and converted to daily scale. However, due to the limited quantity of rainfall during the summer months, when HABs occur, a significant portion of the precipitation data consists of zero values and was therefore omitted from the analysis. In addition, other meteorological factors, except evaporation, were eliminated during modeling experiments since they interact with each other, and evaporation allows for the building of the most accurate model.

#### Data Preprocessing

**Figure 11:**
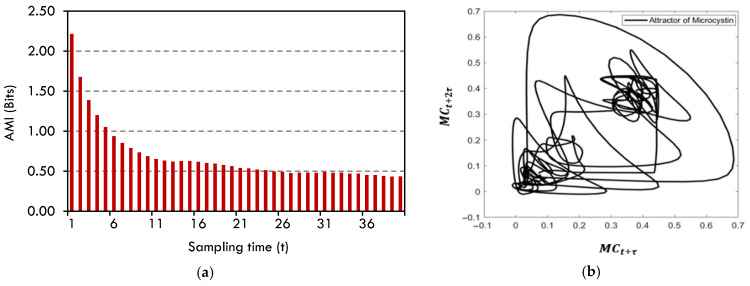
(**a**) Average mutual information (in Bits) (**b**) The attractor of microcystin (MC).

The phase space was reconstructed and then the attractor of microcystin data was plotted to reveal the characteristics of the microcystin data. In order to reconstruct the phase space, it is necessary to determine the time delay and embedding dimension [67]. The study employed the mutual information function [68] to ascertain the time delay. The initial minimal value of average mutual information (AMI) is selected as the optimal time delay. According to Figure 11a, time delay (τ) was taken as 13 and the embedding dimension was assumed to be 3. Figure 11b displays the two-dimensional representation of the resulting attractor projection. Given that the maximal Lyapunov exponent of the Microcystin data is negative (−0.54), it can be concluded that the data is not chaotic [69,70]. However, the presence of a strange attractor in the microcystin data indicates that this data is nonlinear.

**Figure 12:**
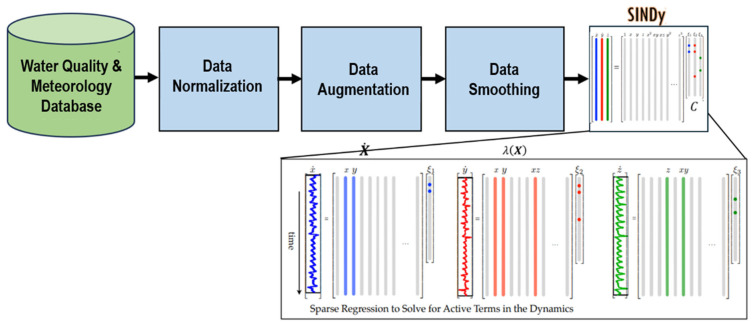
The flow of a sequential process before the application of the SINDy algorithm (Adopted from Brunton et al., 2016 [52]).

Modeling nonlinear data such as microcystin is a challenging task. Furthermore, the presence of measurement mistakes and experimental flaws introduces noise into the data. Deriving the dynamics of a parameter or process from data that is both noisy and nonlinear is an exceedingly intricate undertaking. To ensure accuracy, the microcystin data underwent a sequence of procedures prior to being modeled using the SINDy algorithm (see Figure 12). PySINDy [71,72] was utilized in this study to implement the SINDy application.

**Figure 13:**
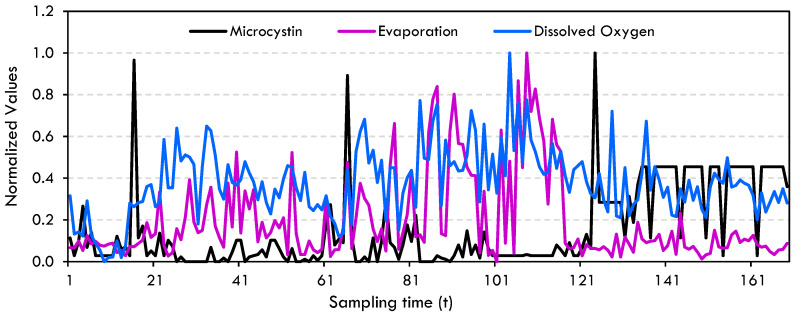
Normalized microcystin, dissolved oxygen and evaporation data.

Data pre-processing techniques, such as standardization and normalization, are used to make variables that have different scales comparable. This helps machine learning algorithms to make more accurate and consistent predictions [56,73,74]. Therefore, microcystin, dissolved oxygen and evaporation values were normalized due to their significant differences in scales. The microcystin data given in Figure 10 is raw data. When we take this data simultaneously with dissolved oxygen and evaporation, it is seen that microcystin data number decreases even more as seen in Figure 13.

**Figure 14:**
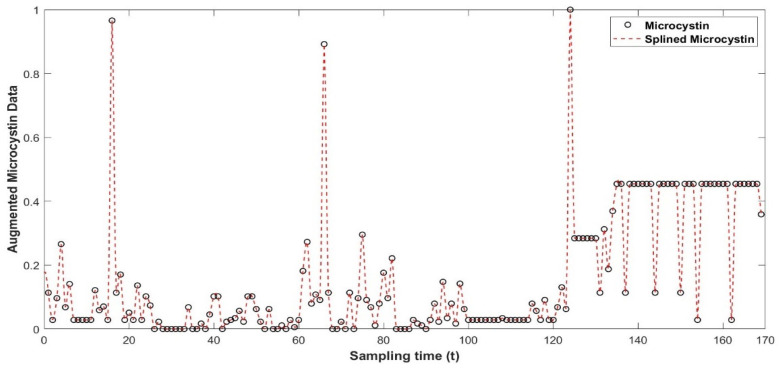
Augmented microcystin data points after modified Akima interpolation.

**Figure 15:**
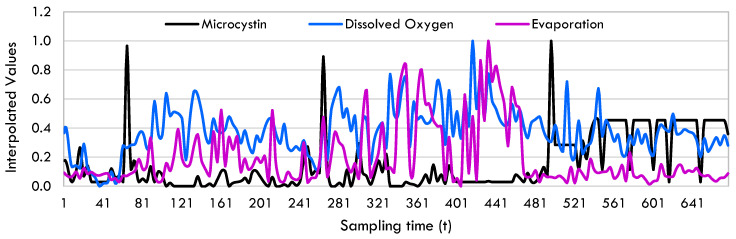
Interpolated microcystin, dissolved oxygen and evaporation data.

The microcystin data utilized in the study were acquired through weekly sampling. In this work, the modified Akima interpolation technique (MAkima) [75], as utilized by [56], was employed due to the need for a finer discretization of the time interval when integrating a continuous-time system of ordinary differential equations and for data augmentation. The MAkima approach incorporates MAkima algorithms and is based on shape-preserving piecewise cubic Hermite interpolating polynomial interpolation (PCHIP) [76]. The authors refer to this pre-processing step as data augmentation due to the increase in the quantity of data points. Interpolation is a data augmentation approach utilized in machine learning systems [77]. Essentially, the MAkima procedure relies on spline interpolation to determine the values between two given points, resulting in a finer level of discretization. Through this procedure, the quantity of data points for each variable is quadrupled. Figure 14 shows the raw and splined microcystin data after the normalization step. Figure 15 shows augmented microcystin, dissolved oxygen and evaporation data together after MAkima interpolation.

**Figure 16:**
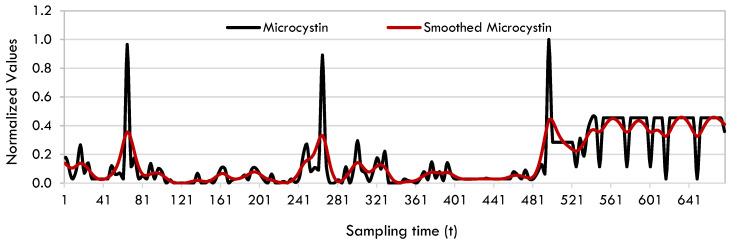
Microcystin and smoothed microcystin data.

The data pre-processing steps have a crucial role in facilitating the extraction of valuable information from data [78]. Applying smoothing and denoising techniques is beneficial for obtaining accurate outcomes when using the SINDy method [79]. In this research, the final stage of data preprocessing involves the process of data smoothing. The Whittaker-Henderson approach [80,81,82,83] was used to smooth microcystin and meteorological variables. Whittaker-Henderson smoothing is a successful method of smoothing discrete-time data that is based on spline smoothing and is specifically designed for equally spaced data points [78]. Figure 16 displays the normalized and augmented microcystin data with the smoothed version of this data. The R libraries utilized for AMI, Lyapunov exponents’ calculations, and Whittaker-Henderson smoothing are ‘tseriesChaos’, ‘nonlinearTseries’, and ‘pracma’, respectively. MatLab was utilized for the implementation of MAkima.

### 4.3. Sparse Identification of Nonlinear Dynamics (SINDy)

Ref. [52] incorporated sparse regression and machine learning with nonlinear dynamical systems to model nonlinear processes using noisy data. The only model structural assumption is that the dynamics are governed by a few key components, thus the equations are sparse in the space of potential functions. Thanks to sparse regression, SINDy identifies the minimal number of terms in the dynamic governing equations necessary for precise data representation. This yields a succinct model that reconciles precision with complexity to prevent overfitting. SINDy is a machine learning technique that derives dynamical system models from time series data, which may manifest as conventional differential equations or partial differential equations [84].

This approach initially constructs a library comprising variations in linear or nonlinear candidate basis functions. Subsequently, the active elements of the coefficients vector are determined by sparse regression. The model is ultimately revised using active terms, while the residual terms are disregarded based on the regularization parameter through sparse regression [85].

State x(t) in a dynamical system can be taken as $\dot{x}=f(x\left(t\right))$. In order to ascertain the function from the data, a temporal evolution of the state $x\left(t\right)$ is collected and either the derivative $\dot{x}(t)$ is measured, or it is numerically approximated from x(t). After sampling the data numerous times and arranging it into two matrices, a data matrix X and its derivative $\dot{X}$ are as follows:

(13) $$X=\left[\begin{array}{c} x^{T}\left(t_{1}\right)\\ x^{T}\left(t_{2}\right)\\ .\\ .\\ .\\ x^{T}\left(t_{m}\right) \end{array}\right]=\left[\begin{array}{c} x_{1}\left(t_{1}\right)\;x_{2}\left(t_{1}\right)\;\ldots \;x_{n}\left(t_{1}\right)\\ x_{1}\left(t_{2}\right)\;x_{2}\left(t_{2}\right)\;\ldots \;x_{n}\left(t_{2}\right)\\ .\;.\;.\\ .\;.\;.\\ .\;.\;.\;\\ x_{1}\left(t_{m}\right)\;x_{2}\left(t_{m}\right)\;\ldots \;x_{n}\left(t_{m}\right) \end{array}\right],\;\dot{X}=\left[\begin{array}{c} {\dot{x}}^{T}\left(t_{1}\right)\\ {\dot{x}}^{T}\left(t_{2}\right)\\ .\\ .\\ .\\ {\dot{x}}^{T}\left(t_{m}\right) \end{array}\right]=\left[\begin{array}{c} {\dot{x}}_{1}\left(t_{1}\right)\;{\dot{x}}_{2}\left(t_{1}\right)\;\ldots \;{\dot{x}}_{n}\left(t_{1}\right)\\ {\dot{x}}_{1}\left(t_{2}\right)\;{\dot{x}}_{2}\left(t_{2}\right)\;\ldots \;{\dot{x}}_{n}\left(t_{2}\right)\\ .\;.\;.\\ .\;.\;.\\ .\;.\;.\;\\ {\dot{x}}_{1}\left(t_{m}\right)\;{\dot{x}}_{2}\left(t_{m}\right)\;\ldots \;{\dot{x}}_{n}\left(t_{m}\right) \end{array}\right]\;$$

A library, denoted as $\lambda \left(X\right)$, is created, which contains potential nonlinear functions of the X.

(14) $$\lambda \left(X\right)=\left[\begin{array}{c} \left|\;|\;|\;\right|\\ \;1\;X\;X^{P_{2}}\;X^{P_{3}}\ldots \\ \;|\;|\;|\;| \end{array}\right]\;$$

$X^{P_{i}}$ denotes polynomials of the ith degree. At this point, a sparse regression problem can be formulated to find a coefficient matrix $C=\xi_{1},\;\xi_{2},\;\ldots ,\;\xi_{n}$ that will identify the active nonlinearities in the dynamic system:

(15) $$\dot{X}=\lambda \left(X\right)C$$

Each column $\varepsilon_{k}$ of C represents a sparse vector of coefficients that determine which terms are active in the right-hand side of one of the row equations ${\dot{x}}_{k}=f_{k}\left(x\right)$ in $\dot{x}=f(x\left(t\right))$. After determining the value of C, a model for each row of the governor equations can be developed in the following manner:

(16) $${\dot{x}}_{k}=f_{k}\left(x\right)=\lambda \left(x^{T}\right)\xi_{k}$$

### 4.4. HALGIS Web Framework

**Figure 17:**
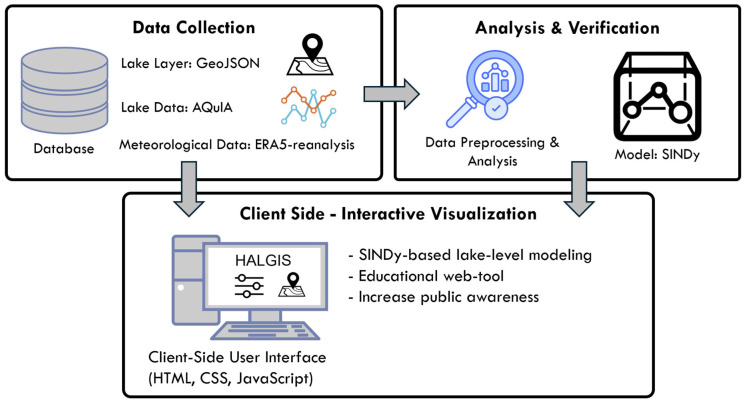
The overall structure and components of web-based framework.

HALGIS, the Harmful ALGae Information System, was developed as a web-based platform to track the formation of harmful algal blooms in Iowa lakes by monitoring the alterations in microcystin levels, a toxin generated by cyanobacteria. HALGIS aims to offer a one-stop digital platform for accessing data and information about the impacts of HABs on public health, recreational activities, and wildlife. The landing page also provides the causes of HAB, information on data integration, analysis, and visualization, and link to data sources. The main stakeholders for HALGIS are the public, students, and environmental education professionals. Therefore, it is crucial to create an interactive and user-friendly interface that is accessible to individuals with limited technical knowledge and expertise. It can be accessed across multiple platforms such as PCs, smartphones, and tablets. HALGIS was organized into multiple layers, as depicted in Figure 17. HALGIS offers data on lakes and HAB conditions to help users comprehend potential HABs and environmental health risks. Users can contribute photos of hazardous lakes using the HALGIS interface.

## Figures and Tables

**Table 1:** Model performance criteria for each lake.

Lake	r	RMSE	MAPE
West Okoboji	0.99	0.0001	1.61
McIntosh Woods	0.69	0.0046	11.3
Blackhawk	0.99	0.0001	1.72
Geode	0.99	0.0047	1.95

**Table 2:** West Okoboji Lake data sources.

Site ID	Site Name
21300001	Gull Point Beach
21300002	Pikes Point Beach
21300003	Triboji Beach
22300009	West Okoboji Lake
14000189	Emerson Bay 1
14000190	Emerson Bay 2
14000191	Emerson Bay 3
14000193	Emmerson T-4
14000410	West Lake Okoboji-Smiths Bay
14000411	West Lake Okoboji-Millers Bay
14000412	West Lake Okoboji-Main Basin North
15300001	Unnamed tributary to Emerson Bay at beach

## Data Availability

The data used in this study is publicly available via the following links: AQuIA Database (The Iowa Department of Natural Resources Water Quality Monitoring and Assessment): https://programs.iowadnr.gov/aquia (Last accessed on 2 July 2025). ERA5-land hourly data: https://doi.org/10.24381/cds.e2161bac.
